# New Onset of Constipation during Long-Term Physical Inactivity: A Proof-of-Concept Study on the Immobility-Induced Bowel Changes

**DOI:** 10.1371/journal.pone.0072608

**Published:** 2013-08-20

**Authors:** Paola Iovino, Giuseppe Chiarioni, Giancarlo Bilancio, Massimo Cirillo, Igor B. Mekjavic, Rado Pisot, Carolina Ciacci

**Affiliations:** 1 Department of Medicine and Surgery, University of Salerno, Salerno, Italy; 2 Division of Gastroenterology of the University of Verona, A.O.U.I., Verona, Italy and Division of Gastroenterology and Hepatology & Center for Functional Gastrointestinal and Motility Disorders, University of North Carolina at Chapel Hill, Chapel Hill, North Carolina, United States of America; 3 Jozef Stefan Institute, Ljubljana, Slovenia; 4 Scientific Research Center, University of Primorska, Koper, Slovenia; Universidad Europea de Madrid, Spain

## Abstract

**Background:**

The pathophysiological mechanisms underlining constipation are incompletely understood, but prolonged bed rest is commonly considered a relevant determinant.

**Aims:**

Our primary aim was to study the effect of long-term physical inactivity on determining a new onset of constipation. Secondary aim were the evaluation of changes in stool frequency, bowel function and symptoms induced by this prolonged physical inactivity.

**Methods:**

Ten healthy men underwent a 7-day run-in followed by 35-day study of experimentally-controlled bed rest. The study was sponsored by the Italian Space Agency. The onset of constipation was evaluated according to Rome III criteria for functional constipation. Abdominal bloating, flatulence, pain and urgency were assessed by a 100mm Visual Analog Scales and bowel function by adjectival scales (Bristol Stool Form Scale, ease of passage of stool and sense of incomplete evacuation). Daily measurements of bowel movements was summarized on a weekly score. Pre and post bed rest Quality of Life (SF-36), general health (Goldberg’s General Health) and depression mood (Zung scale) questionnaires were administered.

**Results:**

New onset of functional constipation fulfilling Rome III criteria was found in 60% (6/10) of participants (p=0.03). The score of flatulence significantly increased whilst the stool frequency significantly decreased during the week-by-week comparisons period (repeated-measures ANOVA, p=0.02 and p=0.001, respectively). Stool consistency and bowel symptoms were not influenced by prolonged physical inactivity. In addition, no significant changes were observed in general health, in mood state and in quality of life at the end of bed rest

**Conclusions:**

Our results provide evidence that prolonged physical inactivity is relevant etiology in functional constipation in healthy individuals. The common clinical suggestion of early mobilization in bedridden patients is supported as well.

## Introduction

Chronic constipation affects up to 30% of people in Western countries with considerable impact on health expenses and quality of life [1]. The pathophysiological mechanisms underlining constipation are incompletely understood. Many strongly held beliefs, such as, that physical activity augmentation may increase bowel frequency, are not evidence-based [1,2]. However, moderate physical activity does not change bowel function [3]; while vigorous physical activity, such as marathon running, seems to increase gut motor activity [3]. In addition, population data support the general notion that physically fit subjects do have a lower incidence of constipation [4,5]. Constipation is a common complaint in older people and the disorder plays a relevant role in hospitalized elderly patients [6–9]. Several studies have suggested that, in elderly patients, increased physical activity is associated with decreased rates of constipation [10,11], whilst a brief period of physical inactivity in normally physically active elderly subjects can prolong colonic transit [12]. Another study, conducted mostly in middle-aged women, suggests that modest physical activity may help only individuals with mild, but not severe constipation [13]. However, the lack of a homogeneous definition of constipation across all these studies makes results difficult to interpret and, may explain discrepancy [2,14]. In addition, sound evidence is lacking and a recent review did not even list prolonged immobilization among the potential determinants of constipation [15]. We might speculate that bowel function may be influenced to some extent by physical activity, but we cannot extrapolate a causative relationship with constipation, so far. It may be that other co-factors such as diet, medications and personality are likely involved in determining both constipation and diminished physical activity.

To our knowledge no studies have been performed in normally physically active healthy individuals to demonstrate whether the isolated decrease of physical activity might modify stool frequency, bowel function and abdominopelvic symptoms leading eventually to a new onset of functional constipation according to an internationally accepted clinical standard (Rome III Criteria for functional gastrointestinal disorders) [16]. Experimentally-controlled bed rest provides an ideal opportunity to study the hypothesized contribution of immobility in developing constipation in healthy individuals, excluding the potential interferences of drugs and comorbidities [17,18].

Our primary aim was to study the effect of long-term physical inactivity experimentally-induced in healthy volunteers on determining a new onset of functional constipation, diagnosed according to the Rome III criteria. Secondary aims were the evaluation of changes in stool frequency, bowel function and abdominopelvic symptoms as well as some measurements of quality of life and general health associated with prolonged physical inactivity.

## Methods

### Study protocol

The study was carried out under the project OSMA (Osteoporosis and Muscular Atrophy) sponsored by the Italian Space Agency in the Valdoltra Hospital, Ankaran, Slovenia. Eligible HV underwent a 7-days run-in (ambulatory period) which was followed by 35-days-6° head-down bed rest (HDBR). Water intake, 24-h urine and faecal excretion, pulse rate (PR) and arterial pressure were recorded daily. Assessment of abdominal symptoms and bowel function, anthropometry and bio-impedance data were collected every week. General health status, quality of life and the presence of depressive symptoms were assessed during the ambulatory period (-1 day before HDBR) and at the end of HDBR (35 days).

### Participants

10 healthy male healthy volunteers (23.2 ± 0.7, range 21-28 yrs) underwent a standardized clinical examination to record their physical data and to exclude: muscular diseases, cardiovascular diseases and unstable body mass index (BMI) during at least the last month before the study. All healthy volunteers had to be physically active and involved in moderate recreational physical activity before the study, but competitive athletes were excluded. They were questioned and scored negative on validated Rome III questionnaires for functional intestinal disorders [16]. In addition, no history of previous abdominal surgery was reported in any subject except for appendectomy.

All subjects signed informed consent before being included in the study, and were aware that they could discontinue their participation at any time. The study fully complied with the Declaration of Helsinki. The study protocol had been approved by the Slovenian National Committee for Medical Ethics at the Ministry of Health.

### Head Down Bed Rest

The study details have been reported elsewhere [18,19]. Briefly, all participants were subjected to the ambulatory period for routine medical screening as well as dietary and environmental adaptation, followed by 6°-HDBR, in which all subjects were kept strictly lying in bed for a period of 35 days, even to perform all daily activities, such as defecation. Adherence to the protocol was ascertained by continuous medical supervision, and also by CCTV. None of them took any medication or underwent any physical or pharmacological countermeasures. Physical activity was not permitted at any time during the period of bed rest. Development of contractures, however, was prevented by passive joint mobilization, which was performed three times a week by a physiotherapist. Participants could watch TV, see movies, read and study. Family and friends could visit them weekly. The diet composition reflected typical Slovenian dietary habits to avoid nutrient composition changes. Six meals (breakfast, lunch, dinner and three snacks) were administered daily. Equally, relative macronutrient content was constantly maintained at 60% of energy from carbohydrate, 25% from fat, and 15% from protein, with a daily fiber intake of 30 grams. Eucaloric diets were tailored to each subject to maintain energy balance throughout the whole study period. Resting energy expenditure was calculated for each individual according to the Food and Agriculture Organization/World Health Organization equations and dietary energy requirements were designed for each subject multiplying resting energy expenditure by 1.4 and 1.2 factors in ambulatory and bed rest conditions, respectively [17,18]. Subjects were required to consume all served food and beverages. Water intake was measured daily and 24-hour urine samples were accurately collected in all the 35 days of the HDBR. Energy balance maintenance was controlled by body composition analysis in terms of fat mass maintenance. Body composition was measured by a non-invasive method to evaluate fat-free mass and fat mass (Human IM plus; DS Dietosystem, Milan, Italy). Bio-impedance and anthropometric measurements were always taken with participants in a supine position. The published results indicated that fat mass did not significantly change throughout the experimental period, while fat-free mass significantly decreased [20]. Mean arterial pressure (MAP) was calculated daily as (Systolic blood pressure - Diastolic Blood pressure)/3 + Diastolic blood pressure.

### Assessment of bowel function and symptoms

A previously published diary to assess bowel function and abdominopelvic symptoms was administered weekly for the duration of the experiment [21,22]. Patients were instructed not to defer for any reason the call to stool, if present. A new onset of functional constipation was evaluated according to the Rome III criteria questionnaire for functional bowel disorders at the end of the study (day 35 HDBR) (Table 1) [16].

**Table 1:** Rome III diagnostic criteria* for functional constipation.

	Must include two or more of the following:
a	Straining during at least 25% of defecations
b	Lumpy or hard stools in at least 25% of defecations
c	Sensation of incomplete evacuation for at least 25% of defecations
d	Sensation of anorectal obstruction/block age for at least 25% of defecations
e	Manual maneuvers to facilitate at least 25%of defecations (e.g., digital evacuation, support of the pelvic floor)
f	Fewer than three defecations per week
2	Loose stools are rarely present without the use of laxatives
3	There are insufficient criteria for irritable bowel syndrome

* Criteria fulfilled for the last 3 months with symptom onset at least 6 months prior to diagnosis

Daily measurement of the number of bowel movements was summarized weekly. Bowel function was assessed on adjectival scales: stool consistency using a validated Bristol stool form scale (a 7-point scale from 1 = separate hard, lumps like nuts to 7 = watery) [23], ease of passage on a previously published adjectival scale (a 7-point adjectival scale ranging from manual disimpaction=1 to incontinence=7) [21] and, the sensation of incomplete evacuation evaluated as subjective perception by a binomial answer (yes or no) [21,22]. The symptoms: bloating, flatulence, abdominal pain and desire to defecate, were assessed on a 100 mm visual analogue scale (VAS).

The adjectival scales for Bristol Stool Form Scale and ease of passage were recorded as numerical values. The proportion of participants with incomplete evacuation was calculated weekly. The VAS symptom scores were rounded to the nearest integer in millimetres.

### General Health Status

General Health Status was assessed by the 12 items-Goldberg’s General Health Questionnaire (GHQ12). The GHQ12 assesses the current mental health. It focuses on two major areas: the inability to carry out normal functions and the appearance of new and distressing experiences. The GHQ12 is a consistent instrument over multiple time periods with relatively long periods between applications in a general population sample as an indicator of minor psychiatric morbidity [24]. The scoring method is a Likert scale scoring method (0-1-2-3) in which the minimum total score is 0 and the maximum GHQ-12 total score is 36. A cumulative score > 32 is considered case positive.

### Assessment of depression mood

The presence of depressive symptoms was evaluated by the Zung self-rating Depression Scale (M-SDS) [25]. The M-SDS a contains 20 items. In the M-SDS score of 44 is the cut-off score for pathological depression.

### Assessment of quality of life

The subjects answered a self-administrated QoL questionnaire, the SF36. This is a generic measure of perceived health status, that incorporates behavioural functioning, subjective well-being, and perception of health by assessing eight health concepts [26]. Physical Component Summary (PCS) and Mental Component Summary Scales (MCS) were also calculated [27]. The results of a patient’s SF-36 form for each item was compared with those of Italian Control Groups and of US population [27,28].

### Statistical Analysis

The primary endpoint for analysis in this study was the effect of 35-days of physical inactivity on new onset of functional constipation using Rome III criteria. The primary analysis focused on change of the presence/absence of functional constipation before and at the end of experimental bed rest using McNemar’s exact test. The secondary endpoints included bowel function scores and severity VAS scores for bowel symptoms.

The secondary analysis focused on contrasting week-by-week water intake, 24h-urine volume, MAP, PR, anthropometry, the bowel function scores (frequency, consistency, proportion of weeks with the sense of incomplete evacuation) and the severity of individual symptoms (bloating, flatulence, abdominal pain and desire of defecation) during the bed rest period using a repeated-measures ANOVA model. The M-SDS, GHQ12 and SF36 scores were compared within groups with paired t test or Wilcoxon signed rank test for paired comparisons samples, as warranted. Spearman’s correlation (Rs) was used as appropriate. Data are presented as mean ± SE, unless otherwise indicated; Significance was expressed at the p<0.05 level. The SPSS software package for Windows (release 15.0.1; SPSS Inc, Chicago, IL, USA) was used for statistical analysis.

## Results

The demographics of participant are shown in Table 2.

**Table 2:** Demographic details of healthy participants.

	**Mean**	**SE**
**Men (n)**	10	
**Weight (Kg)**	74.7	3.0
**BMI (Kg/m^2^)**	23.3	0.6
**% fat body mass**	16.5	1.2
**Lean body mass (Kg)**	31.8	1.1

**Figure 1:**
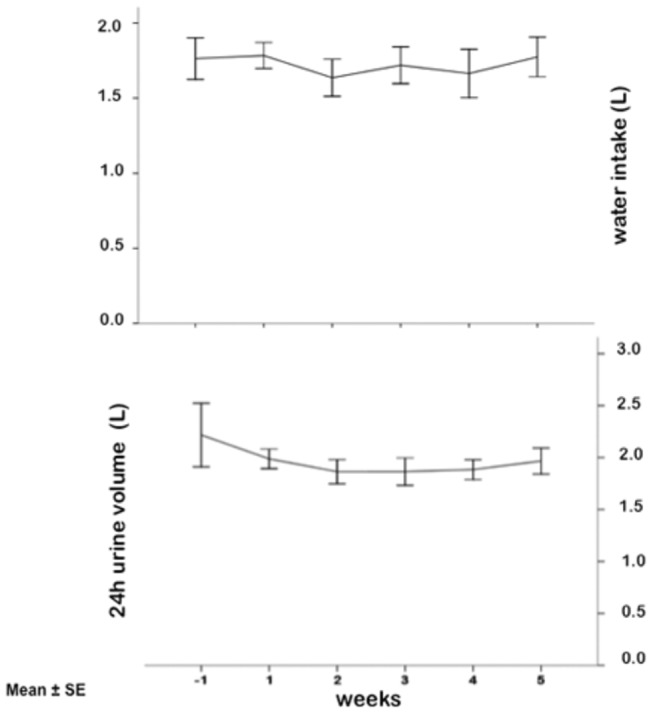
Water intake and 24-h urine volume during ambulatory and bed rest period in 10 male healthy volunteers. ANOVA for repeated measures demonstrated no significant changes during week-by-week comparisons.

**Figure 2:**
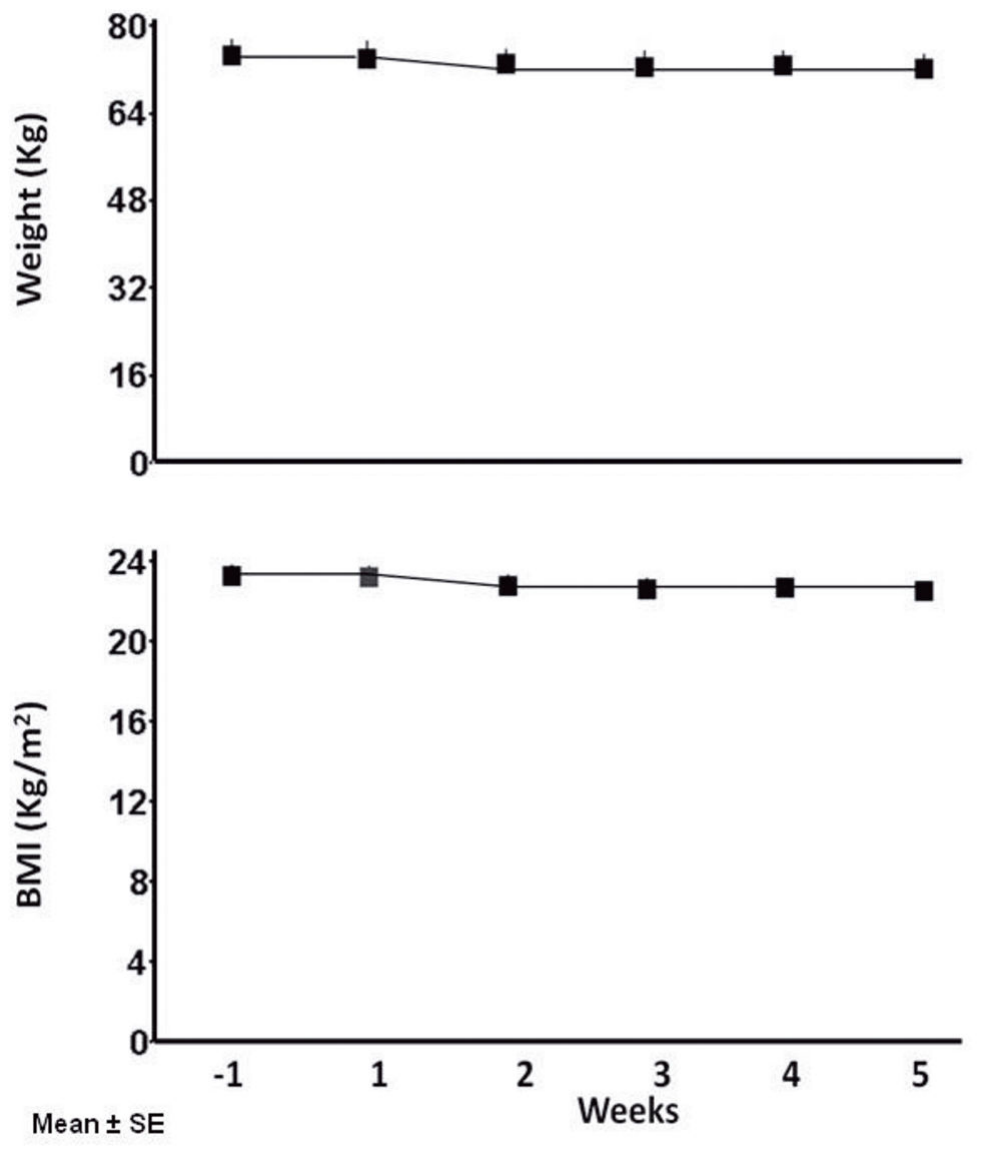
Weight and Body Mass Index (BMI) of 10 male healthy volunteers (mean±SE) significantly decreased during week-by-week comparisons period (ANOVA for repeated measures, p<0.001 and p=0.002, respectively).

**Figure 3:**
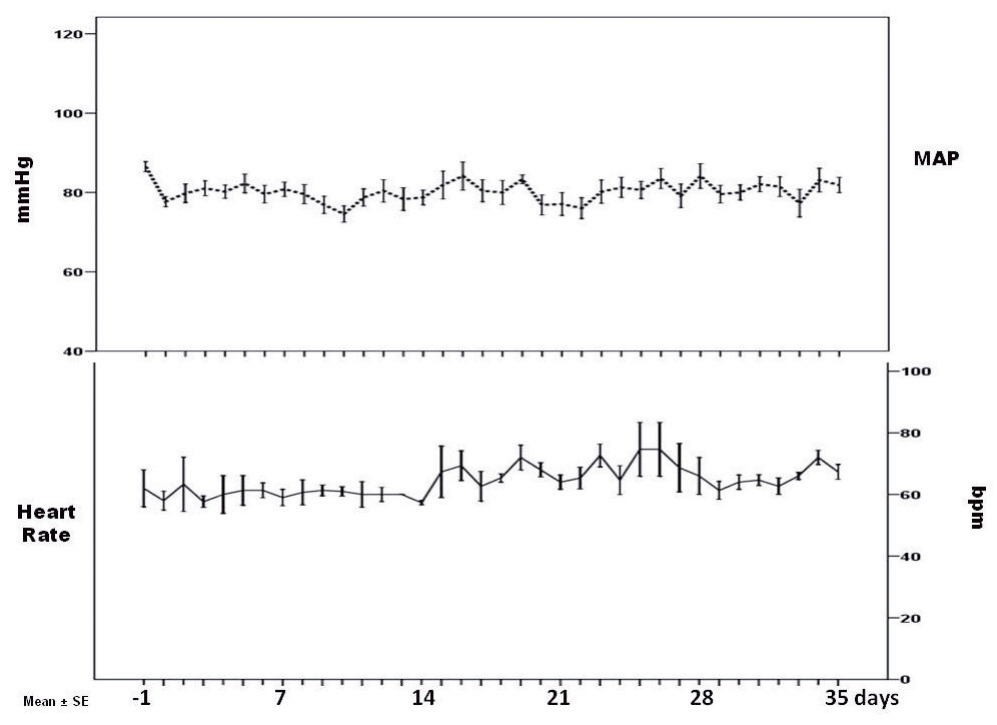
Mean Arterial Pressure (MAP) and pulse rate (PR) significantly change along the experiment in 10 male healthy volunteers, p < 0.001and p<0.001, respectively (mean±SE).

All participants completed the study in good health and attended all scheduled appointments. Participants did not modify water intake and the 24-h urine volume remained unchanged during ambulatory and bed rest period (repeated-measures ANOVA, p=0.42 and 0.5, respectively) (Figure 1). Weight and BMI significantly decreased during the week-by-week comparisons period from baseline (Figure 2). MAP and PR significantly change along the experiment (Figure 3).

### Bowel symptoms and function

#### Primary outcome

New onset of functional constipation fulfilling Rome III criteria was found in 60% (6/10) of participants at the end of the study (day 35 HDBR) (p=0.03).

#### Secondary outcome

**Figure 4:**
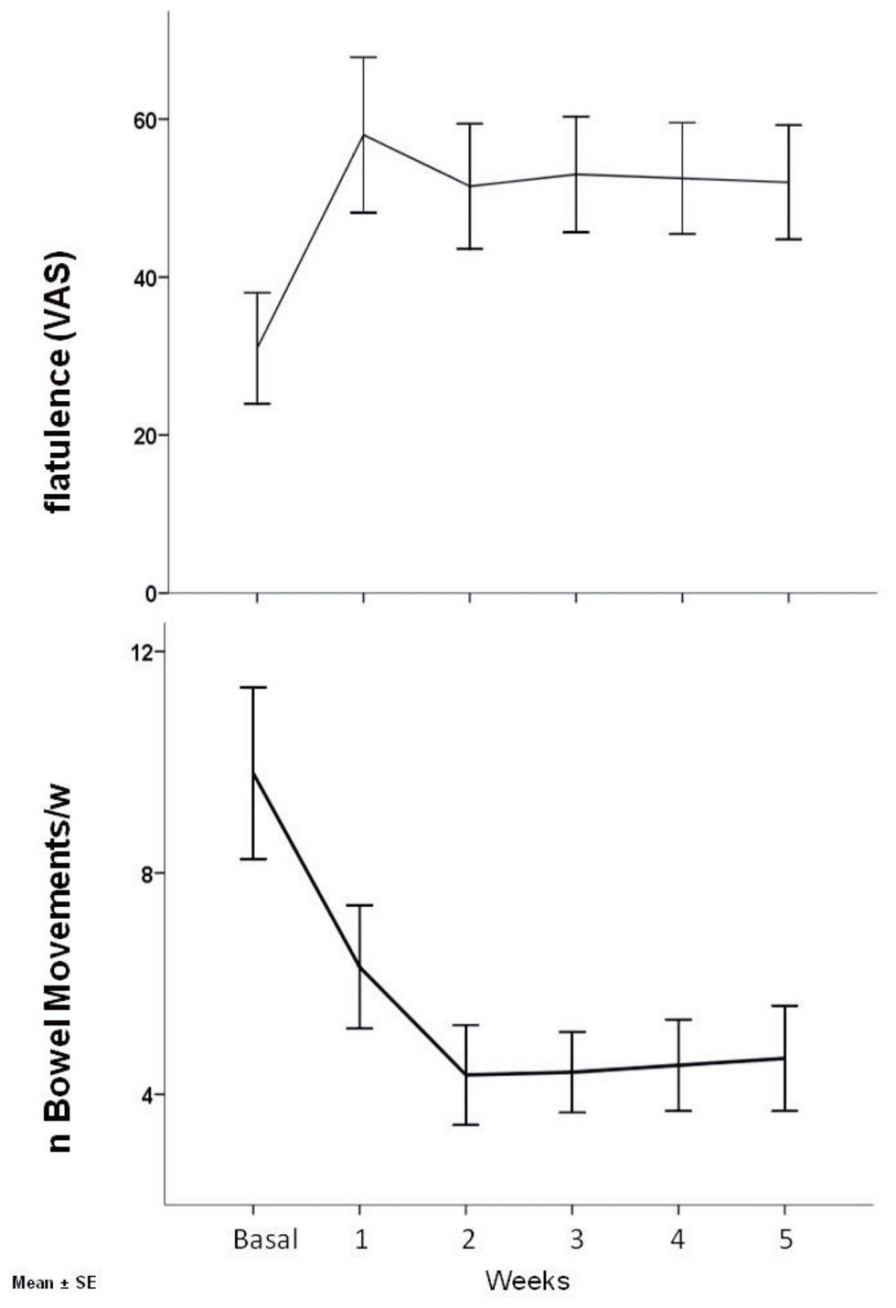
VAS scores of flatulence and number of bowel movements during ambulatory and bed rest period in 10 male healthy volunteers (mean±SE) (Repeated-measures ANOVA, p=0.02 and p=0.001, respectively).

The score of flatulence significantly increased, whilst the stool frequency significantly decreased during the week-by-week comparisons period (p=0.02 and p=0.001, respectively) (Figure 4). A significant correlation was found between the change of flatulence score (baseline to end of bed rest) and the change in stool frequency (baseline to end of bed rest) (Rs=0.90, p<0.001). There were no statistically significant differences detected for any other parameters studied (Table 3).

**Table 3:** Analysis of bowel function scores during ambulatory period and bed rest period. Values are expressed as Mean ± SE.

	**BLOATING(0-100)**	**PAIN (0-100)**	**DESIRE TO DEFECATE(0-100)**	**STOOL FORM (1–7)**	**EASE OF PASSAGE (1–7)**	**(%) WITH INCOMPLETE EVACUATION**
**AMBULATORY PERIOD**	5.0 ± 2.7	0.5 ± 0.5	16.5 ± 8.8	3.3 ± 0.2	3.7 ± 0.2	0 ± 0
**Week 1**	15.5 ± 7.1	6.0 ± 6.0	14.5 ± 8.3	3.3 ± 0.2	3.3 ± 0.1	0 ± 0
**Week 2**	3.0 ± 2.1	2.0 ± 2.0	22.0 ± 8.4	3.3 ± 0.4	3.6 ± 0.3	10 ± 10
**Week 3**	16.5 ± 8.0	4.0 ± 4.0	20.0 ± 7.3	3.0 ± 0.4	3.4 ± 0.3	10 ± 10
**Week 4**	9.3± 4.3	0.0 ± 0.0	26.8 ± 5.3	3.0 ± 0.3	3.3 ± 0.2	30 ± 15
**Week 5**	2.0 ± 2.0	0.0 ± 0.0	33.5 ± 6.9	3.0 ± 0.2	3.3 ± 0.2	20 ± 13
**ANOVA** for repeated measures	p= 0.13	p= 0.57	p= 0.23	p= 0.67	p= 0.51	p= 0.45

### General Health

There were no significant differences in the GHQ 12 cumulative scores before and at the end of the bed-rest period (13.1±0.9 vs 12.1±0.3, p=0.3). No subjects scored below 32 which is the cumulative score chosen to identify a case.

### Quality of life

**Figure 5:**
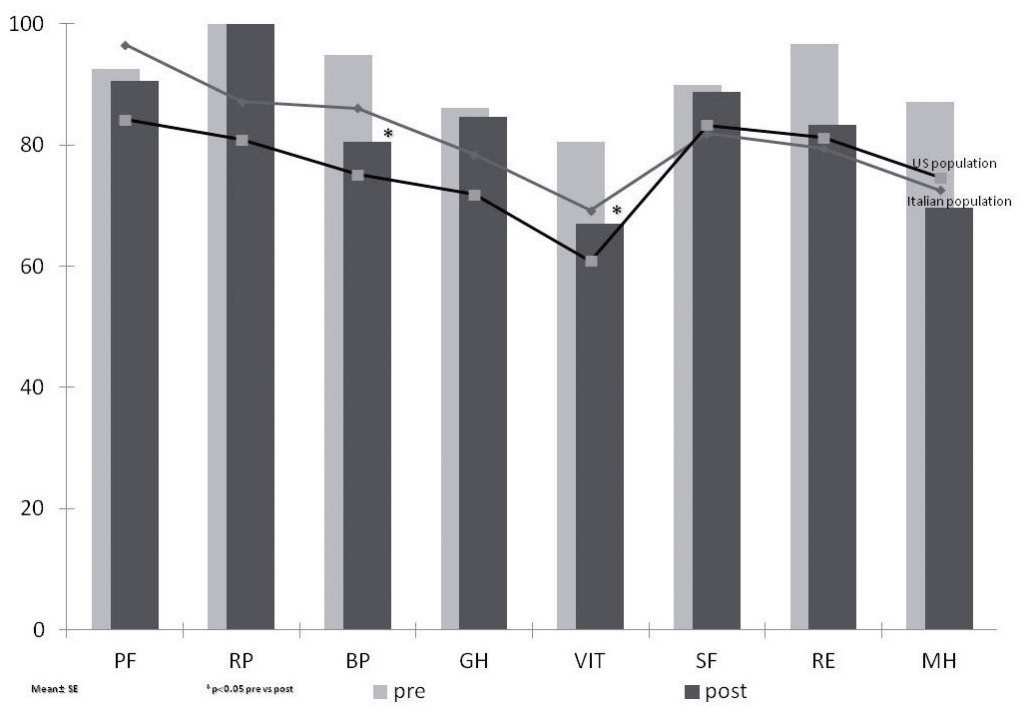
The mean SF-36 scale scores of 10 male healthy volunteers (mean±SE). The scores on all scales range from 0 to 100, with higher scores reflecting better health. * p <0.05 pre- vs postbed rest using paired t-test.PF (physical function), (RP) role-physical, (BP) bodily pain, (GH) general health, (VT) vitality, (SF) social function, (RE) role-emotional, (MH) mental health.

Figure 5 shows the 8-item SF36 scores for all subjects before and at the end of the bed rest period. Although none of the participants showed any significant difference in the 8-items compared to Italian Control Group and US population matched for age and sex both before and at the end of the bed rest period, bodily pain and vitality domains significantly decreased at the end of the bed rest. The summary score of SF-36 PCS and MCS did not show any significant difference before and at the end of the bed rest period (PCS 55.6±1.2 vs 55.9 ±2.7- MCS 55.9 ±0.8 vs 48.6 ±4.3, p=0.9 and p=0.1, respectively). At the end of the bed rest period only 2 subjects (n=5 and n=8) showed respectively MCS and PCS < 40, which is considered the cut-off score [27].

### Zung Depression Mood State

None of the participants fulfilled criteria for pathological depression before HDBR. At the end of HDBR only two subjects (n=5 and n=9) showed M-SDS score ≥ 44. No significant difference was found before and at the end of the bed rest period (31.1±1.9 vs 34.8±3.4, p=0.22). Excluding from the analysis the two subjects with M-SDS score ≥ 44 and/or subjects with PCS or MCS< 40 at the end of the bed rest period the results do not change. No correlation was found between participants with new onset of functional constipation and either participants with positive M-SDS score (Rs=-0.61, p=0.06) or with a positive PCS (Rs =-0.41, p=0.24) and MCS score (Rs=-0.41, p=0.24).

## Discussion

The novel finding of our study was that prolonged bed-rest rest may determine a new onset of functional constipation according to Rome III criteria. Moreover, among the secondary outcomes flatulence severity score significantly increased, while stool frequency significantly decreased.

Functional constipation is a commonly reported disorder with increasing prevalence in older age where its clinical relevance seems also to increase [29]. In fact, in the hospitalized patients its management seems to influence the duration of the hospital stay [30]. In the elderly, chronic constipation is significantly associated with lower urinary tract symptoms and, the medical relief of constipation improves lower urinary tract symptoms [31]. In addition, constipation may rarely lead to a stercoraceous perforation of the colon, a life threatening disease [32]. Physical inactivity seems to be a relevant etiologic factor for constipation, but scientific evidence to this regard is scanty [9]. However, there is some evidence that sitting hospitalized elderly patients out of bed for long periods may be detrimental to their recovery [33]. In addition, the commonly given physicians’ advice that increasing physical exercise is beneficial to large bowel motor function seems sound common sense, but poor when it comes to scientific evidence even in young subjects [2,14].

Head down bed rest in healthy subjects is a suitable model to investigate the net effects of inactivity on colon function excluding interferences of pharmachological treatments and comorbidities, particularly common in the elderly [17–19]. To our knowledge, no studies have been performed using this approach, which allows observing changes in stool frequency, bowel function and symptoms closely associated with physical inactivity. This study protocol has been designed to prevent potential interferences of changes in fluids intake, diet, fiber amount, and any modification in total energy balance. Therefore, we are confident that the observed changes are not secondary to diet modifications. Bio-impedance data confirmed the expected results on fat-free mass [17], and actually fat mass did not change [20].

Our results showed a new onset of functional constipation at the end of 35-days of bed rest in 60% of healthy volunteers as diagnosed by Rome III criteria. However, no participant fulfilled Rome III criteria for a new-onset of constipation-predominant Irritable bowel syndrome.

A recent study, performed in patients after stroke without preexisting constipation, reported similarly a new-onset constipation on the basis of Rome II criteria in 55% of patients in the first 4 weeks. However, in hospitalized post-stroke patients other factors (i.e. constipating drugs) might contribute to the onset of constipation.

Our study took into account a number of bowel function parameters to better define which of them are associated with prolonged physical inactivity suggesting an etiology of constipation. The stool consistency, measured by the validated Bristol stool form scale, is an objective dimension of stool form observation and seems correlated to colonic transit times in healthy volunteers [34]. Keeping in mind, that this scale may not reliably gauge the severity of delayed transit in constipated patients [36], a significant modification of stool consistency was not reported by our healthy volunteers making an acquired colon motility disorder unlikely [34,35]. A significant reduction in stool frequency was found in our study, but a poor correlation was found between stool frequency and colonic transit time [23,35] then, stool frequency remains a poor clinical surrogate for objectively measured whole-gut and colonic transit time. Another risk factor for constipation in this study was the obliged lying position during the experimental period that requested a bedpan usage. It is common knowledge that also normal subjects when provided with a sensation of stooling may show a dyssinergic pattern of defecation, particularly when tested in left lateral position [36]; Consequently, unfavorable environment negatively affects the defecation, possibly together with the inhibition of the desire to defecate, and may lead to constipation [37,38]. It has been previously suggested that anorectal disfunction underlying functional constipation may be an important contributory factor to the development of bloating and abdominal distension [39]. In our study there was a significant increase in flatulence and a significant correlation between the change in flatulence that significantly increased and the change in number of evacuations that significantly decreased. It has been previously demonstrated that in healthy subjects rectal distension experimentally-induced accelerates gas evacuation and prevents gas retention [40]. In fact, intestinal gas handling is a dynamic and efficient process that allows the normal gut to accommodate and evacuate large gas loads without inducing symptoms [40]. It is possible that during bed rest due to an ineffective evacuation of stools, a larger amount of faeces were retained in the rectum, then causing rectal distension and explaining the decreased bowel frequency and the increase in desire of defecate along the experimental period. Consequently an increase in flatulence was observed. Concerning this, interestingly, mild physical activity and upright position accelerate gas transit and reduce bloating and abdominal distension [41,42]. Theoretically, a change in hydrostatic forces distribution and increased intra-abdominal pressure during upright position or a propulsive gut motor response induced by exercise, promote gas transit.

At the end of the bed rest period a participant (n=5) showed both M-SDS score and MCS score of SF36 below the cut off value, while 2 other participants showed an abnormal M-SDSand the other in PCS of SF36. Analyses repeated excluding these 3 participants did not change the results pre-post bed rest. No correlations were found with each scale score and the new onset of constipation and no participant with new onset of functional constipation had positive M-SDS, PCS or MCS scores. There are few previous studies that demonstrate an increase of depression mood during shorter bed rest, but these studies were performed in different populations, mainly Japanese, and using different bed rest protocol, which might explain the different results.

### LIMITATION OF THE STUDY

We acknowledge the limitations of this study: the overall sample size is small, restricted to a special setting and the study population consists only of same aged men. However, this might be an advantage to investigate the net effects of inactivity excluding also interferences of sex hormones, particularly progesterone, that seems to play a role in the development of constipation [43]. Unfortunately, in our study no assessment of colonic transit time was allowed for X-rays exposure risk. Moreover, we were not allowed to perform the diagnostic tests needed to diagnose functional defecation disorders according to Rome III Criteria, although these tests would be helpful to better explain the pathophysiological mechanisms underlying the new onset of functional constipation. In fact, there was a concern about including invasive procedures in this observational study.

In conclusion, our study provide evidence of prolonged bed rest as relevant etiology for functional constipation in healthy volunteers. Determining mechanism/s is/are left unclear but the increment in flatulence reported by the subjects supports indirect evidence of a potentially acquired functional defecation disorder. In addition, these results provide the missing scientific basis to implement suggestions of early mobilization in bedridden patients in order to prevent the onset of constipation. Further studies on a larger number of healthy individuals are warranted to better define the net effect of physical activity on bowel motor function. 
